# Identification of CDC20 as a Novel Biomarker in Diagnosis and Treatment of Wilms Tumor

**DOI:** 10.3389/fped.2021.663054

**Published:** 2021-08-26

**Authors:** Qinlin Shi, Bo Tang, Yanping Li, Yonglin Li, Tao Lin, Dawei He, Guanghui Wei

**Affiliations:** ^1^Ministry of Education Key Laboratory of Child Development and Disorders, Chongqing Key Laboratory of Pediatrics, Chongqing Key Laboratory of Children Urogenital Development and Tissue Engineering, China International Science and Technology Cooperation Base of Child Development and Critical Disorders, Pediatric Research Institute, Children's Hospital of Chongqing Medical University, Chongqing, China; ^2^Department of Pediatric Urology Surgery, Children's Hospital of Chongqing Medical University, Chongqing, China

**Keywords:** Wilms tumor, biomarkers, cell division cycle 20, diagnosis, cell proliferation

## Abstract

**Objective:** Wilms tumor (WT) is a common malignant solid tumor in children. Many tumor biomarkers have been reported; however, there are poorly targetable molecular mechanisms which have been defined in WT. This study aimed to identify the oncogene in WT and explore the potential mechanisms.

**Methods:** Differentially expressed genes (DEGs) in three independent RNA-seq datasets were downloaded from The Cancer Genome Atlas data portal and the Gene Expression Omnibus database (GSE66405 and GSE73209). The common DEGs were then subjected to Gene Ontology enrichment analysis, protein–protein interaction (PPI) network analysis, and gene set enrichment analysis. The protein expression levels of the hub gene were analyzed by immunohistochemical analysis and Western blotting in a 60 WT sample. The univariate Kaplan–Meier analysis for overall survival was performed, and the log-rank test was utilized. A small interfering RNA targeting cell division cycle 20 (CDC20) was transfected into G401 and SK-NEP-1 cell lines. The Cell Counting Kit-8 assay and wound healing assay were used to observe the changes in cell proliferation and migration after transfection. Flow cytometry was used to detect the effect on the cell cycle. Western blot was conducted to study the changes of related functional proteins.

**Results:** We commonly identified 44 upregulation and 272 downregulation differentially expressed genes in three independent RNA-seq datasets. Gene and pathway enrichment analyses of the regulatory networks involving hub genes suggested that cell cycle changes are crucial in WT. The top 15 highly connected genes were found by PPI network analysis. Furthermore, we demonstrated that one candidate biomarker, CDC20, for the diagnosis of WT was detected, and its high expression predicted poor prognosis of WT patients. Moreover, the area under the curve value obtained by receiver operating characteristic curve analysis from paired WT samples was 0.9181. Finally, we found that the suppression of CDC20 inhibited proliferation and migration and resulted in G2/M phase arrest in WT cells. The mechanism may be involved in increasing the protein level of securin, cyclin B1, and cyclin A

**Conclusion:** Our results suggest that CDC20 could serve as a candidate diagnostic and prognostic biomarker for WT, and suppression of CDC20 may be a potential approach for the prevention and treatment of WT.

## Introduction

Wilms tumor (WT) is a common pediatric solid retroperitoneal tumor. The incidence of WT was ~6 per 100,000 to 7 per 100,000 for children younger than 15 years (1, 2). Thanks to the continuous efforts by the Children's Oncology Group and the National Wilms Tumor Society (NWTS), the overall survival rate of WT has improved from 30 to 90% in the last 30 years (3). However, some cases still result in poor outcomes, which is associated with metastasis, recurrence, anaplastic WT, and chemoradiotherapy resistance (4). Moreover, chronic health conditions secondary to treatment impact nearly one quarter of survivors of WT and include renal failure, infertility, cardiac toxicity, restrictive pulmonary disease, and the development of subsequent malignancies (5, 6). Hence, finding a novel strategy for the diagnosis and treatment of WT has become a hotspot in recent years. Most research on WT biomarkers has focused on the genetic components of WT development including WT1, WTX, MYCN, CTNNB1, SIX1/SIX2, TP53, loss of heterozygosity 11p15, 16q, and 1p and 1q gain of function (7–9). A recent whole-exome study has identified that DROSHA and DICER1 mutations impair expression of tumor-suppressing miRNAs (10). Unfortunately, the frequency of alterations in genes is similarly uncommon, and there is no clear gene for clinical application (11).

The Gene Expression Omnibus (GEO) is an international public repository that archives and freely distributes microarray, next-generation sequencing, and The Cancer Genome Atlas (TCGA) is a large-scale cancer genome project that provides researchers with multidimensional maps of the key genomic changes (12, 13). Both GEO and TCGA have significantly increased our understanding of cancer. Therefore, in this study, we first identified the common differentially expressed genes (DEGs) from multiple microarrays and TCGA WT RNA-sequence dataset. The upregulation DEGs were then subjected to Gene Ontology (GO) enrichment analysis, protein–protein interaction (PPI) network analysis, and gene set enrichment analysis (GSEA). According the bioinformatics results, one candidate biomarker, cell cycle 20 (CDC20) (cell division cycle 20 homolog, also called Fizzy), was performed to detect the expression level in 60 paired WT samples. Receiver operating characteristic (ROC) analysis and Kaplan–Meier (KM) analysis were performed to identify diagnostic and prognosis markers for WT. In addition, we predicted and verified the effect of knockdown of CDC20 on WT cell lines. CDC20 small interfering RNA (siRNA) can knock down CDC20 expression at protein levels and thereby lead to cell cycle arrest in the G2/M phase in WT cells. Taken together, the present findings provide more valuable strategies for the diagnosis and treatment of patients with WT.

## Materials and Methods

### Study Population

RNA-sequence data for WT patients were downloaded from the TCGA data portal (https://tcga-data.nci.nih.gov/tcga/) and the GEO database (GSE66405 and GSE73209, http://www.ncbi.nlm.nih.gov/geo), which contains 184 WT tissues and 12 adjacent non-tumor tissues. The TCGA Target-WT sample clinic data were downloaded using package ‘TCGAbiolinks' in R.

### DEG Analysis

GEO database (GSE66405 and GSE73209) and TCGA database analyses of DEGs between WT and their non-tumor counterparts were performed using package “DESeq2” in R. The DEGs were screened using *p* < 0.05 and |logFC| > 1.5 as the thresholds. Next, heatmaps and volcano plots based on the upregulated and downregulated genes in each dataset were plotted using the “pheatmap” and “ggplots” package of R software. Then, the downregulated and upregulated genes on the three databases were intersected using the “gridBase” and “VennDiagram” package of R software.

### GO Enrichment Analysis

GO enrichment analysis was performed using Database for Annotation, Visualization, and Integrated Discovery (DAVID; http://david.abcc.ncifcrf.gov/). The DAVID tool was used for obtaining the enriched GO terms of differentially expressed mRNA genes based on the hypergeometric distribution to compute values, which was described in a previous study (14). The enriched biological processes (BPs), cellular component (CC), and molecular function (MF) were obtained to analyze the common DEGs at the functional level. *p* < 0.05 was set as the threshold value.

### PPI Network Construction and Pathway Analysis

STRING (Search Tool for the Retrieval of Interacting Genes/Proteins, http://string-db.org/) is a biological database and Web resource of known and predicted PPIs. Based on the STRING database, PPIs of DEGs were selected with a score (median confidence) of >0.7, and the PPI network was then visualized by Cytoscape (http://www.cytoscape.org/). The hub protein was selected based on its association with other proteins. The DEGs with more association with other DEGs indicate important roles in the PPI network. In addition, the CDC20 single GSEA was performed using the “clusterProfiler,” “ggplots” R package of R software.

### Patient Tissue

We obtained WT tissues and adjacent kidney tissues from 60 patients who underwent surgery for WT at the Department of Urology Surgery of the Children's Hospital of Chongqing Medical University from January 2015 to January 2020. All specimens were histopathologically identified as WT, and all WT tissues were classified according to the American National Wilms Tumor Study 5 (NWTS-5) typing and TNM staging system by pathologists at the Children's Hospital of Chongqing Medical University who were blinded to the results. After the specimens were extracted, they were placed immediately in liquid nitrogen and for further examination by immunohistochemical (IHC) analysis and Western blotting (WB) experiments.

### Cell Lines and Cell Culture

The human Wilms cell lines (G401 and SK-NEP-1) were purchased from the American Type Culture Collection. Both SK-NEP-1 and G401 cells were maintained in McCoy 5A medium (Sigma-Aldrich, Shanghai, China) and supplemented with 15% fetal bovine serum (FBS) and 1% penicillin/streptomycin (Gibco, NY, USA); the cells were cultured at 37°C in a humidified atmosphere with 5% CO_2_.

### siRNA Transfection

Three segments of CDC20 siRNA and a negative control (NC) were synthesized and purified by Guangzhou RuiBo Company (Guangzhou, China). Target sequences for siRNAs were ACCAACCCAUCACCUCAGUtt ACUGAGGUGAUGGGUUGGUtt (CDC20 si1), GGAGCUCAUCUCAGGC-CAU ttAUGGCCUGAGAUGAGCUCC tt (CDC20 si2), and CAAGAAGGAA-CAUCAGAAA tt UUUCUGAUGUUCCUUCUUG tt (CDC20 si). The G401 and SK-NEP-1 cells were plated onto 6- or 12-well plates and transiently transfected using Lipofectamine™ RNAiMAX (Invitrogen, USA) according to the manufacturer's protocol.

### Cell Proliferation and Migration

Cell Counting Kit-8 (CCK-8) assays (Dojindo, Japan) were performed to determine cell proliferation. Approximately 1 × 10^4^ G401 or SK-NEP-1 cells were seeded into 96-well plates and transfected with si-CDC20-1, si-CDC20-2, si-CDC20-3, or NC oligonucleotides. At the indicated time points (hours 0, 24, 48, and 72), the culture medium was removed, and 100 μL of CCK-8 medium was added to each well. The cells were incubated for an additional 4 h, and the optical density was measured at an absorbance wavelength of 450 nm on a microplate reader (Bio-Rad, USA).

Wound healing assays were used to evaluate cell migration. Briefly, G401 cells were seeded in 6-well plates and incubated for 24 h, followed by transfection with an si-CDC20-1, si-CDC20-2, si-CDC20-3, or NC oligonucleotides. Then, scratching was performed with 10-μL pipette tips when the cell confluence reached 100%. Next, the cells were washed several times with phosphate-buffered saline (PBS) to remove the floating cells, and the medium was replaced with fresh cell culture medium without FBS. Images were taken of non-overlapping fields in each well at 0, 24, and 48 h after the scratching step using ImageJ software (http://imagej.en.softonic.com).

### Cell Cycle Analysis

The transfected cells were detached by EDTA-free trypsin (Gibco, NY, USA), washed with precooled PBS, and fixed in 75% ethanol at 4°C overnight. The cells were resuspended in 0.2 mL of PI/RNase Staining Buffer (BD Biosciences, Shanghai, China) and incubated in the dark for 30 min. The cells were analyzed using a flow cytometer (BD Biosciences).

### Immunohistochemistry

Imunohistochemistry studies were performed on formalin-fixed, paraffin-embedded WT and adjacent tissue sections obtained from untreated patients with WT according to standard procedures. Briefly, 4-μm-thick paraffin sections were deparaffinized and rehydrated, and antigen retrieval was performed. Then, the sections were incubated with 3% H_2_O_2_ and 0.5% bull serum albumin (BSA). The primary antibodies used were a CDC20 rabbit antibody (1:200, Absin, Shanghai, China). Histochemistry score [H score = ∑ (PI × *I*) = (percentage of cells of weak intensity × 1) + (percentage of cells of moderate intensity × 2) + (percentage of cells of strong intensity × 3)] (15) was obtained with Quant Center Analysis tool.

### Western Blot

Total protein was extracted from tissues and transfected cells using radioimmunoprecipitation assay lysis buffer (Beyotime, China) supplemented with phenylmethanesulfonyl fluoride, and the concentrations were determined by bicinchoninic acid assay. Following protein extraction, sodium dodecyl sulfate–polyacrylamide gel electrophoresis was performed. Then, the electrophoretic bands were transferred to polyvinylidene fluoride membranes (Millipore, USA). Next, the membranes were incubated in 5% BSA (ZSGB-BIO, Beijing, China)–Tris-buffered saline with Tween 20 for 1 h. We used a CDC20 rabbit antibody (1:1,000, Absin, China), securin (1:5,000, Abcam, Shanghai, China), cyclin B1 (1:3,000, Abcam, USA), and cyclin A (1:2,000, Abcam, USA) and GAPDH mouse antibody (1:800, ZSGB-BIO, China) as primary antibodies. After incubating the membranes with primary antibodies and the corresponding secondary antibodies, we detected positive bands with a chemiluminescent reaction. Image collection and densitometry analysis were executed with Quantity One (Bio-Rad, Shanghai, China).

### Statistical Analysis

The KM analysis for overall survival proceeded based on the gene's expression level, the cutoff level of which was set at the median value with the aid of GraphPad Prism 7 software and the log-rank test was utilized. One-way analysis of variance and two-tailed Student *t*-tests were used for expression data comparisons by using GraphPad Prism 7 software. Each experiment was repeated three times or more, and all data were presented as mean ± standard deviation (SD). Statistical significance was described as follows: #*p* > 0.05, not significant; ^*^p ≤ 0.05; ^**^p ≤ 0.01; ^***^p ≤ 0.001; ^****^p ≤ 0.0001.

## Results

### The DEGs Among GSE66405, GSE73209, and TCGA

**Figure 1:**
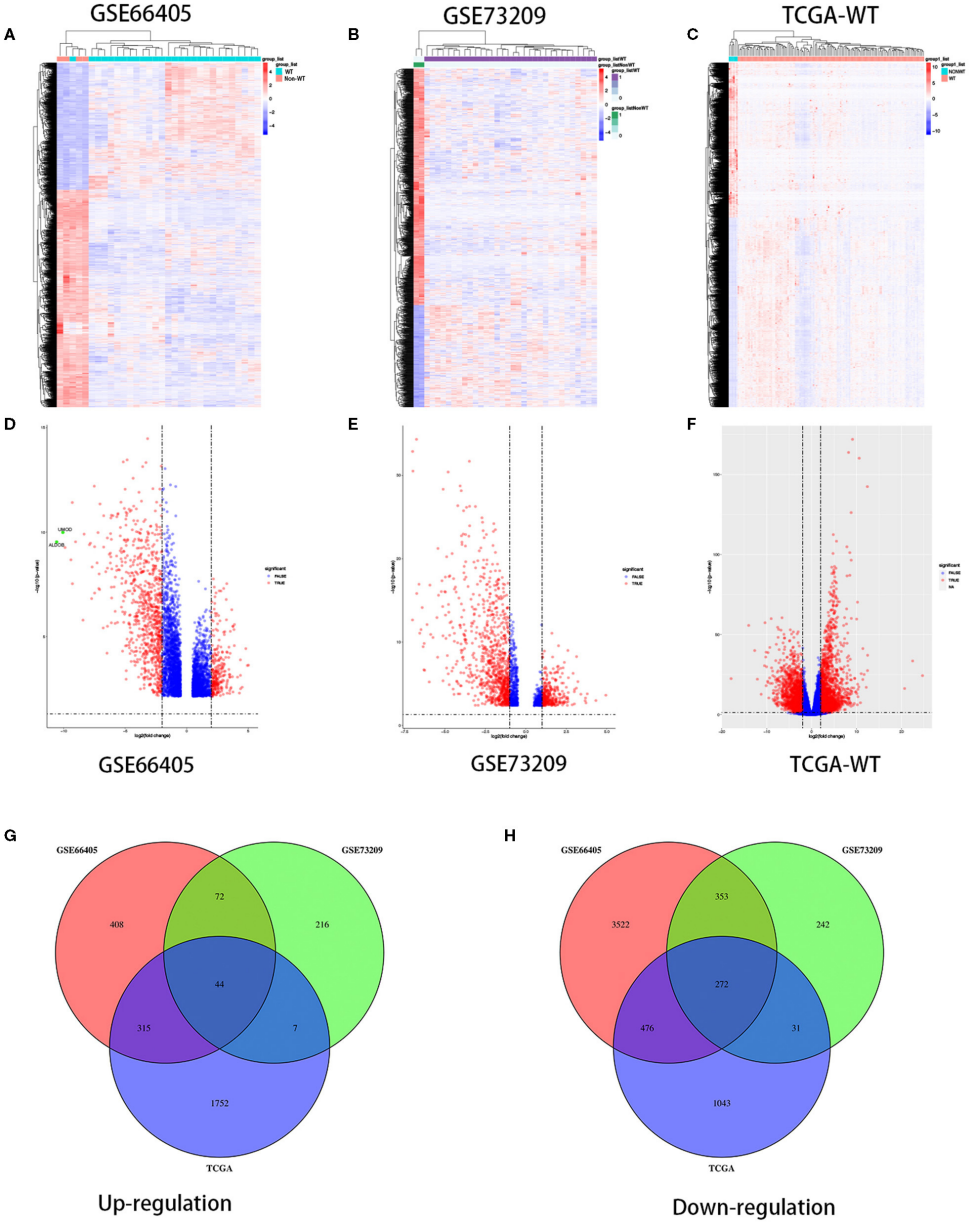
Differentially expressed genes in three independent datasets. Heatmaps of the DEGs in the WT gene expression datasets GSE66405, GSE73209, and TCGA-WT, respectively **(A–C)**. Volcano plots of genes that are significantly different between WT tissues and normal controls in datasets GSE66405, GSE73209, and TCGA-WT, respectively **(D–F)**. X axis indicates the fold change (log-scaled), whereas the Y axis shows the *p*-values (log-scaled). Each symbol represents a different gene, and the red color of the symbols categorizes the upregulated/downregulated genes falling under different criteria (*p* value and fold-change threshold). *p* < 0.05 is considered as statistically significant, whereas fold change = 1.5 is set as the threshold **(D–F)**. The common differentially expressed genes among GSE66405, GSE73209, and TCGA **(G,H)**.

To determine the different mRNA expression profiles in WT, our study performed three mRNA microarray analyses of 184 WT tissues and 12 non-tumor adjacent tissues (Figures 1A–C). As the volcano plots illustrated, gene expression profiles from GSE66405 identified 5,462 DEGs with 839 genes upregulated and 4,623 genes downregulated in WT samples compared with the non-tumor adjacent tissues (Figure 1D). From GSE73209 data, we recognized 1,237 DEGs, of which 339 genes were upregulated and 898 genes were downregulated in WT (Figure 1E). We identified 3,940 differentially expressed mRNAs, including 2,118 upregulated mRNAs and 1,822 upregulated mRNAs from TCGA database (Figure 1F). We identified 44 commonly upregulated genes and 272 downregulated genes in the above datasets via Venn diagram (Figures 1G,H).

### GO and Pathway Enrichment Analysis

**Figure 2:**
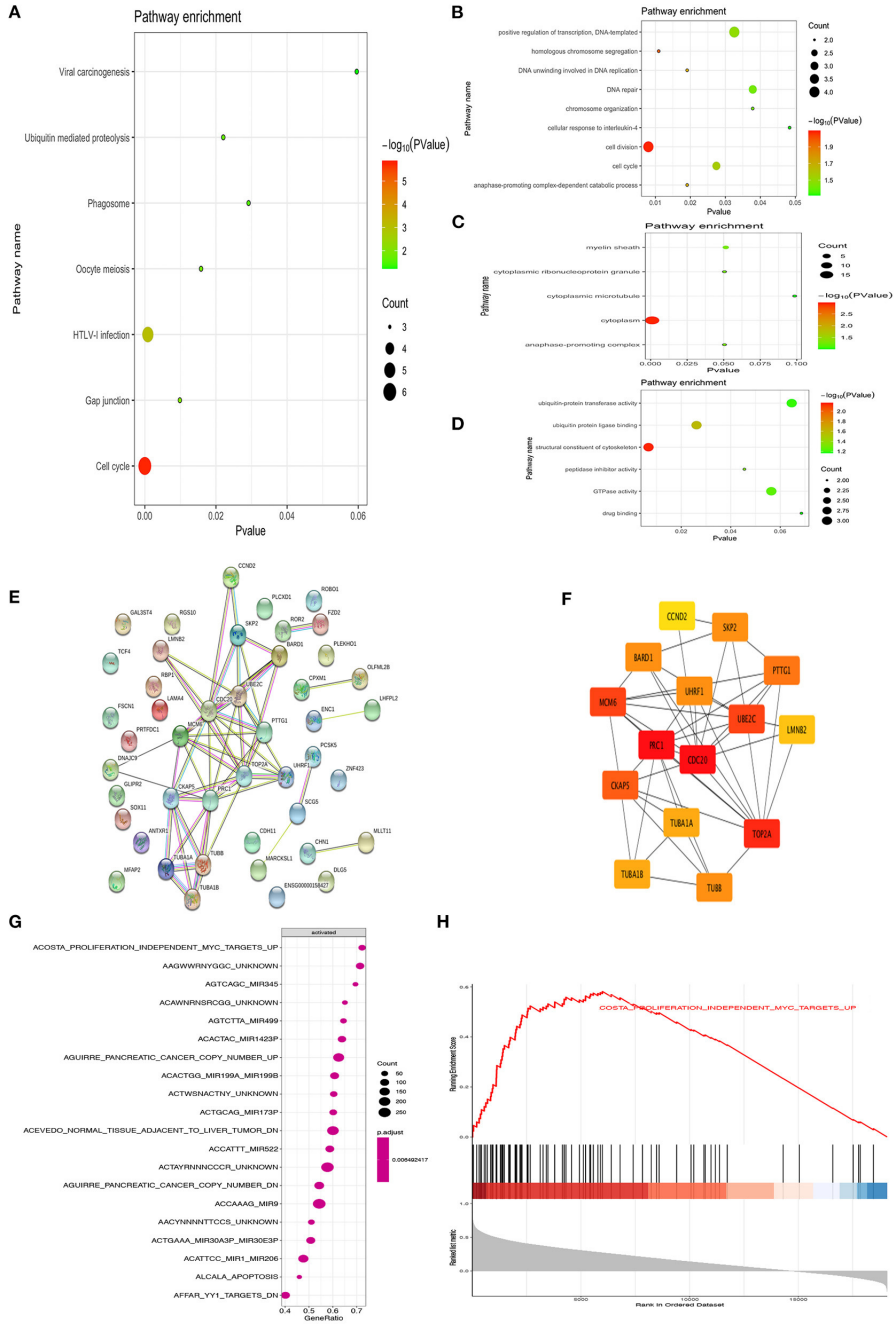
Bioinformatics analysis of different genes. Gene ontology analyses of the common up-regulation DEGs according to biological process, cellular component and molecular function **(A–D)**. PPI network of the common DEGs identified from GSE66405, GSE73029, and TCGA was constructed **(E)**. The sub-networks were identified by Cytoscape MCODE plugin **(F)**. Gene set enrichment analysis of CDC20 related genes from TCGA datasets **(G,H)**.

DAVID was used to analyze the Kyoto Encyclopedia of Genes and Genomes (KEGG) pathway and GO analysis of 44 common upregulation genes. The KEGG disease enrichment analysis demonstrated that targets were associated with the cell cycle, HTLV-I infection, oocyte meiosis, phagosome, gap junction, ubiquitin-mediated proteolysis, and viral carcinogenesis (Figure 2A). The GO analysis showed that, for BPs, genes significantly enriched in cell cycle, cell division, positive regulation of transcription, DNA template, homologous chromosome segregation, DNA unwinding involved in DNA replication, DNA repair, anaphase-promoting complex-dependent catabolic process, chromosome organization, and cellular response to interleukin 4 (Figure 2B). For MF, genes were primarily enriched in drug-binding ubiquitin–protein transferase activity, ubiquitin protein ligase binding, structural constituent of cytoskeleton, peptidase inhibitor activity, GTPase activity, and drug binding (Figure 2C). For CC, genes were particularly enriched in the cytoplasm, anaphase-promoting complex, cytoplasmic ribonucleoprotein granule, myelin sheath, and cytoplasmic microtubule (Figure 2D).

### Key Candidate Genes Identification With DEG PPI Network

The PPI network of DEGs was constructed by using the STRING online database and Cytoscape (Figure 2E). MCODE plugin was used for module analysis of the PPI network, and the most significant modules were chosen for further pathway analyses based on the degree of importance. Then, the central node genes (more than 10 connections/interactions) were identified, and the top 15 highly connected genes were TOP2A, PTTG1, SKP2, TUBB, TUBA1A, UHRF1, TUBA1B, UBE2C, CDC20, CCND2, BARD1, MCM6, CKAP5, LMNB2, and PRC1 (Figure 2F). The genes in the module were mainly associated with increased cell cycle, cell division, cell cycle process, regulation of cell cycle, mitotic cell cycle process, and G2/M transition of mitotic cell cycle. As previously reported, CDC20 is an oncogene that plays a crucial role in cell cycle, cell division, and cell process (16, 17). Hence, we further investigated the role of CDC20 in WT. Furthermore, we applied single GSEA on the TCGA dataset and found that CDC20 was mainly regulated by MYC, Mir-345, Mir-449, Mir-1423P, Mir-199A/B, Mir-522, Mir-9, and Mir-206 (Figure 2G). Moreover, MYC is significantly correlated with CDC20 (Figures 2G,H).

### Expression of CDC20 Was Higher in WT Tissues Compared With Adjacent Normal Tissues

**Figure 3:**
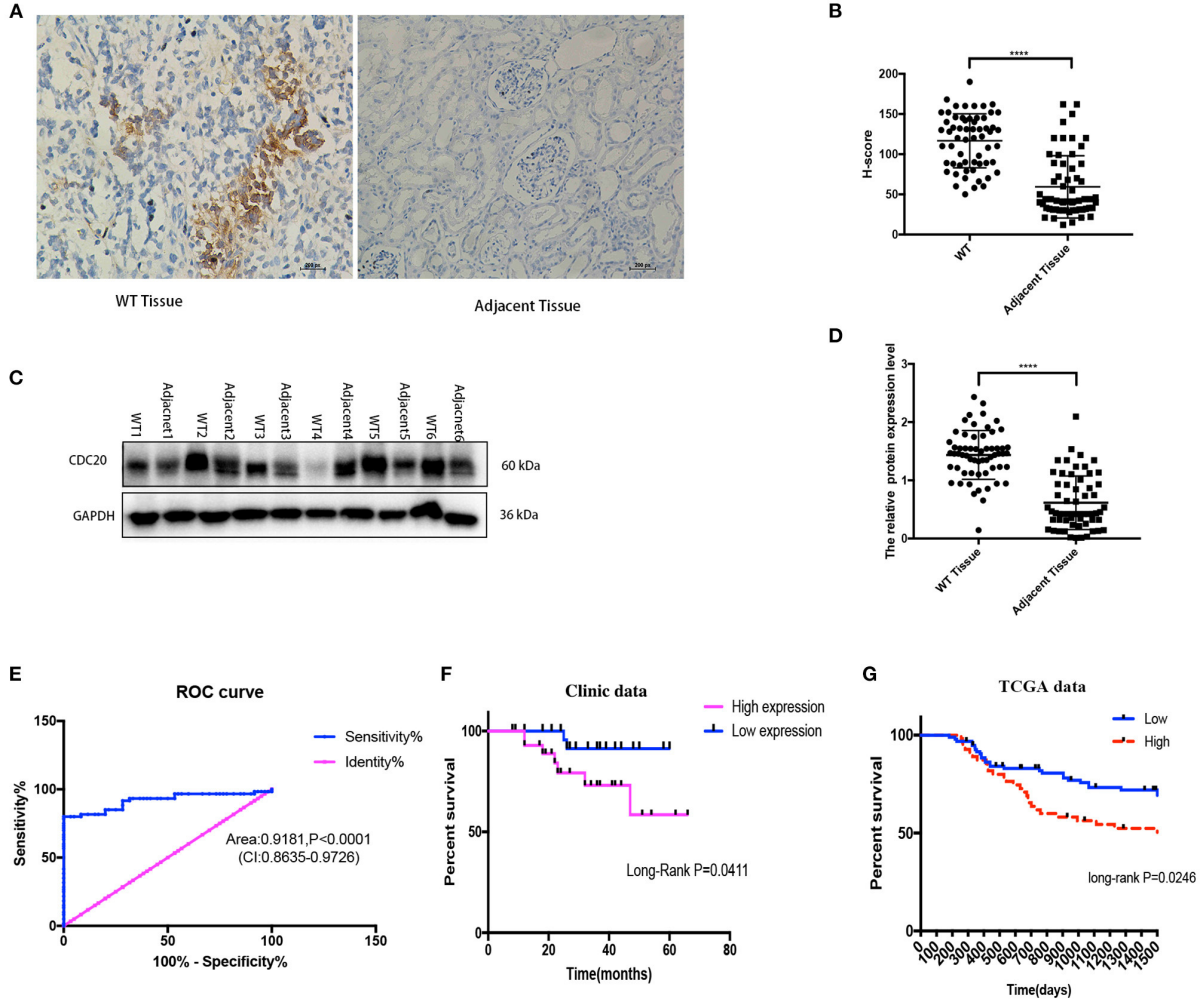
CDC20 expression is upregulated in clinical WT samples. Representative images of IHC staining for CDC20 in adjacent tissues and different histologic grades of WT tissues **(A)**. The protein levels of CDC20 in six pairs of WT tissues and adjacent non-tumor tissues measured by Western blot **(C)**. Quantification of CDC20 IHC staining and Western blot in paired WT and adjacent tissues, respectively **(B,D)**. Receiver operating characteristic curve from IHC staining shows CDC20 is a marker to distinguish WT tissues from adjacent tissues **(E)**. Kaplan–Meier analysis of overall survival was performed to indicate that higher expression of CDC20 was correlated with poor survival of WT patients **(F,G)**. *p*-values were obtained from the log-rank test. ****p ≤ 0.0001 were obtained by Student *t*-test. All data are represented by mean ± SD.

In order to verify the results of the above bioinformatics analysis, WB (quantitative) and imunohistochemistry (semiquantitative and localization) methods were used for examination expression of CDC20 in WT clinic samples. The protein expression of CDC20 was detected by WB and CDC20 staining, and the results were similar to the IHC result in WT tissues. In IHC staining, H score revealed that CDC20 was significantly highly expressed in WT tissues compared with paired adjacent normal kidney tissues (*p* < 0.0001, Figures 3A,B). WB results, which were similar to IHC results, revealed high expression in WT tissues compared with paired adjacent normal kidney tissues (*p* < 0.0001, Figures 3C,D). In order to sequence the CDC20 diagnostic sensitivity, the area under the curve value obtained by ROC curve analysis from paired WT samples was 0.9181, which held statistical significance to support the diagnostic value of CDC20 for WT (Figure 3E). Furthermore, to detect the relationship between high expression of CDC20 and clinical prognosis, we used the KM survival analysis and log-rank test. Interestingly, we found that the high expression of CDC20 (median value) had a markedly lower overall survival rate. The results were similar in our clinical samples and in the TCGA database (log-rank *p* < 0.05, Figures 3F,G). Altogether, these data implied the potential oncogenic role of CDC20 in WT, and high expression of CDC20 may influence the survival rate of WT patients.

### CDC20 Promotes WT Cell Proliferation and Migration and Controls Cell Cycle Progression *in vitro*

**Figure 4:**
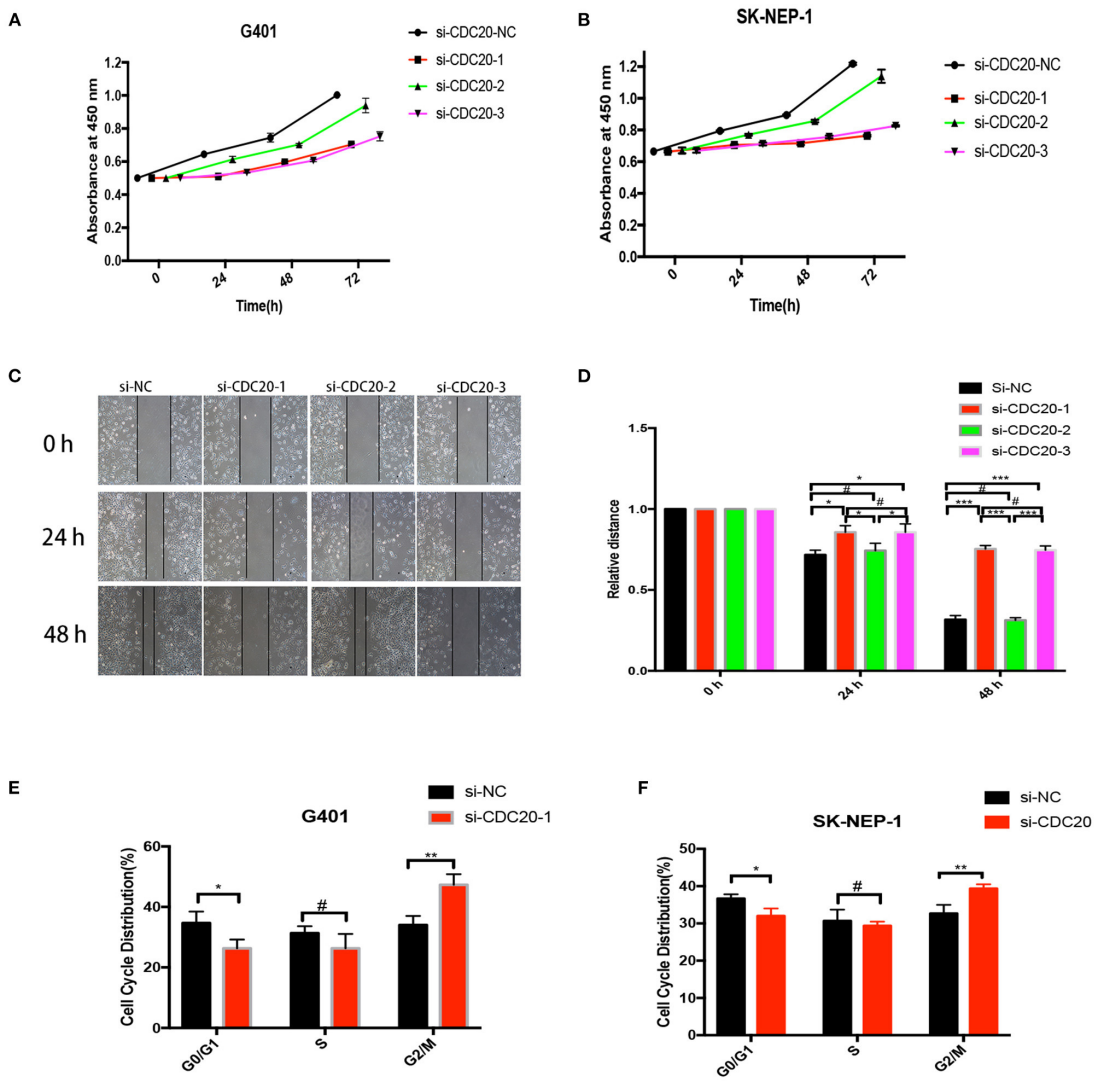
CDC20 controls cell proliferation, migration, and cell cycle *in vitro*. CDC20 siRNA suppression of proliferation of G401 and SK-NEP-1, respectively, *in vitro* **(A,B)**. Wound healing assays were performed to determine the migration rate of G401 cells at 24 and 48 h after transfection of siRNA **(C,D)**. The G0/G1, S, and G2/M phase proportions of G401 and SK-NEP-1 cells transfected with siCDC20 or NC **(E,F)**. Results are shown as the mean ± SD. For comparisons, the Student *t*-test was performed. *p < 0.05, **p < 0.01, ***p < 0.001, #*p* > 0.05.

As in the results mentioned previously, CDC20 may be involved in the tumorigenesis of WT. However, the potential mechanism is unknown. To explore whether CDC20 can be used as a new strategy for the treatment of WT, three silenced RNA segments were used for CDC20 in G401 and SK-NEP-1 WT cell lines. We performed CCK-8 assays to examine the proliferation effect of si-CDC20 WT cells. As determined by the CCK-8 assay, si-CDC20-1 and si-CDC20-3 significantly slowed cell proliferation in a time-dependent manner in G401and SK-NEP-1 cells compared with the cells transfected with NC siRNA and siCDC20-2 (*p* < 0.001, Figures 4A,B). These results indicate that si-CDC20 could decrease WT cell proliferation. In addition, we used wound healing assays to examine the migration ability after downregulation of CDC20. Compared with the si-NC, the si-CDC20-1 and CDC20-3 could significantly impair the migration of G401 cells lines in 24 and 48 h (*p* < 0.05 and *p* < 0.001, respectively; Figures 4C,D). Next, the cell cycle distribution was altered by the si-CDC20-1 in SK-NEP-1 and G401 cell lines. Compared with the si-NC, the proportion of G0/G1 phase cells was significantly decreased in SK-NEP-1 and G401 (*p* < 0.05, Figures 4E,F). On the contrary, the proportion of G2/M phase cells was reduced by si-CDC20 in SK-NEP-1 and G401 cell lines (*p* < 0.01, Figures 4E,F). Based on these data, we hypothesized that downregulation of CDC20 may inhibit proliferation and migration by inducing cell cycle arrest in G2/M phase.

### Cell Cycle–Related Proteins Levels Were Suppressed by Inhibition of CDC20 in WT Cells

**Figure 5:**
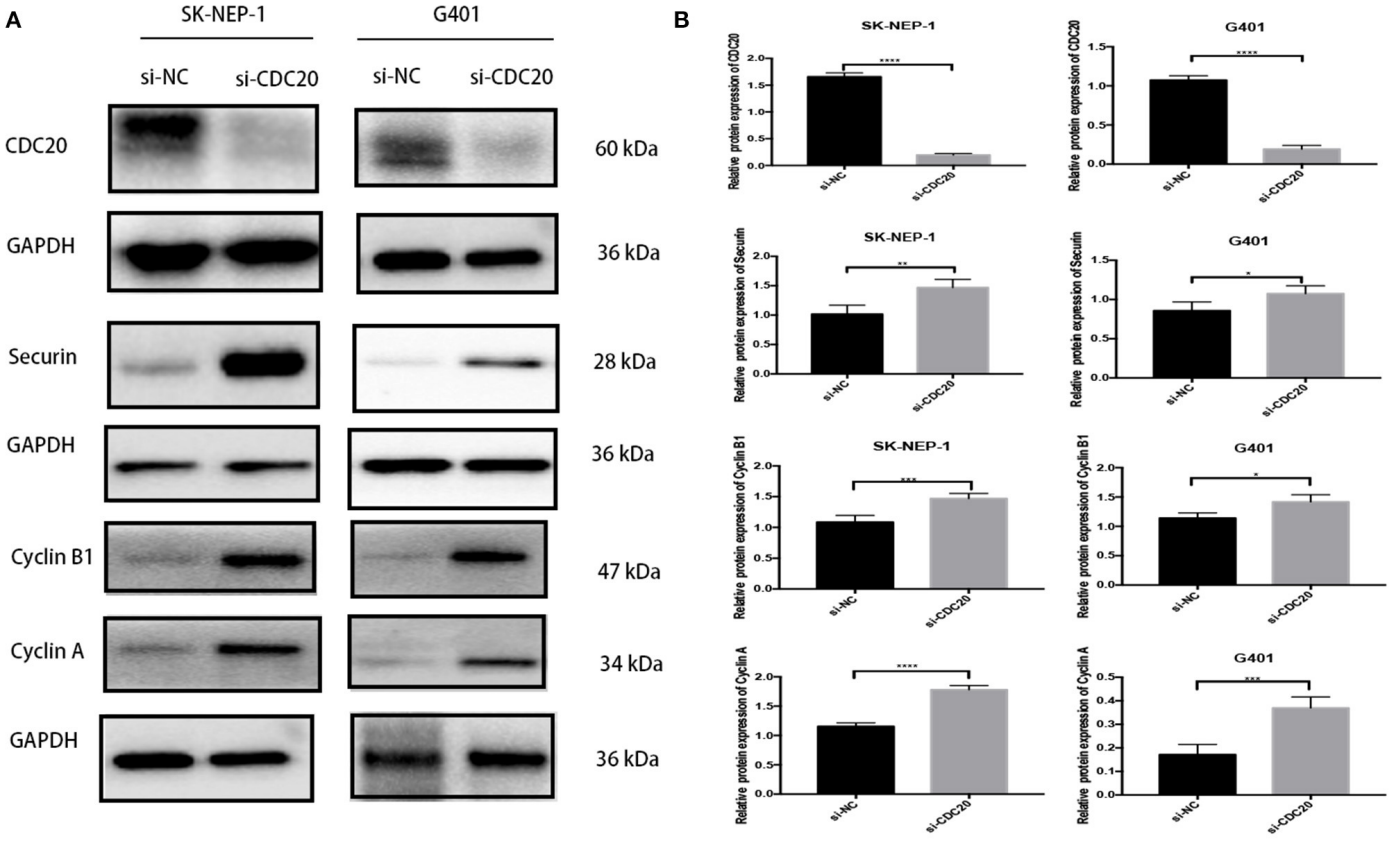
Western blot analyses of proteins that regulate G2-to-M transition in WT cells lines inhibiting CDC20. Suppression of CDC20 protein expression by siRNA in SK-NEP-1 and G401 cells. Western blot analysis of CDC20, securin, cyclin B1, and cyclin A protein levels of the siCDC20 group compared with the NC group in SK-NEP-1 and G401 cells **(A)**. Quantification of CDC20, securin, cyclin B1, and cyclin A Western bolt in SK-NEP-1 and G401 cell lines, respectively **(B)**. Analysis was performed by Western blotting 72 h after the siRNA transfection. Results are shown as the mean ± SD. For comparison, the Student *t*-test was performed. *p < 0.05, **p < 0.01, ***p < 0.001, ****p < 0.0001.

It has been previously reported that CDC20 plays an important role during the metaphase-to-anaphase transition by targeting critical cell cycle regulators including securin and cyclin B1 and cyclin A for ubiquitination-mediated destruction (18–20). In addition, in human malignant tumors, inhibition of CDC20 in growing cells leads to G2 arrest with a consequent decrease of cyclin B1, securin, and cyclin A (21). In the study, the WB results showed that protein expression level of CDC20 could be significantly inhibited by si-CDC20 in SK-NEP-1 and G401 cell lines (Figures 5A,B). Meanwhile, compared with the si-NC group, the expression levels of securin and cyclin B1 and cyclin A were markedly decreased in the si-CDC20 group (Figures 5A,B), supporting the results of the cell cycle analysis. Taken together, the aforementioned findings suggest that silence of CDC20 arrests the cell in G2/M phase of WT cell.

## Discussion

The carcinogenesis of WT involves many factors that lead the cells to undergo uncontrolled proliferation (22). However, the underlying molecular mechanisms remain unclear. A recent study showed eight genes (EGF, CDK1, ENDRA, NGFR, OIP5, NUF2, and CDCA8) are predicted to be involved in carcinogenesis pathways (23). But, the study involved only TCGA dataset which did not represent a generalization. Moreover, the study did not exclude 6 metastatic specimens according to TCGA nomenclature principles. More importantly, TARGET dataset included only unfavorable histology WT cases that relapsed and anaplastic WT cases, indicating that this dataset is not a representative random sampling of WT but rather a highly selected set. In this study, we have further identified common significant DEGs from three independent studies. The PPI network of DEGs revealed the top 15 highly connected genes, and CDC20 plays a crucial role in WT as the node connecting core. Functional analyses demonstrated that these DEGs are mainly associated with the cell division and cell cycle process. Meanwhile, many studies showed that CDC20 plays an oncogenic role in human tumorigenesis. Overexpression of CDC20 was observed in a variety of human tumors including pancreatic cancer, breast cancer, prostate cancer, lung cancer, colorectal cancer, hepatocellular carcinoma, glioblastoma, gastric cancer, and other types of human cancer (24–27). Therefore, CDC20 is usually identified as an oncogene (16). A recent study identified that nine key genes including CDC20 were potential diagnosis genes in clear cell renal cell carcinoma (28). Meanwhile, the study by Gayyed et al. showed that high expression of CDC20 was associated with high tumor grade in RCC (29). However, there are no further studies on the relationship of high expression of CDC20 between WT and RCC. Although both WT and RCC occur in the kidney, the difference is that WT originates in embryonic cells, and more than 95% of WTs occur in children. Moreover, an early study showed that CDC20 expression in RCC may be involved in cytochrome P450 1B1 (CYP1B1) (30).

In this study, we assessed the expression level of CDC20 in 60 paired WT tissues and corresponding non-tumor samples. The results indicated that the protein level of CDC20 in Wilms tumor tissues was much higher than that in matched nontumor tissues. Immunohistochemistry was used to investigate the subcellular location of CDC20 and its relationship with clinical pathological parameters of WT patients. By ROC analysis, we found that the high expression of CDC20 may provide diagnostic value in paired WT samples. In addition, by KM analysis and log-rank test, we found that higher CDC20 protein expression level was associated with poor survival rate. To investigate the potential biological function and molecular mechanism of CDC20 in WT, we designed a double-stranded, siRNA targeting CDC20 to interfere with its expression level in the WT cell lines.

By cellular proliferation assay, migrate assay, and fluorescence-activated cell sorting test, we found that cells transfected with siCDC20 oligonucleotides showed decreased growth speed, a reduced rate of migration, and an increased proportion of cells in the G2/M stage. The specific knockdown of CDC20 by siRNA showed a suppressed effect against WT cell proliferation and migration *in vitro*, which indicated that the overexpression of CDC20 might be expected to accelerate cell proliferation and promote tumor initiation and progression of WT. The WT cells with suppressed CDC20 expression were induced to accumulate in the G2/M phase, which may be responsible for the inhibition of cell growth. Taken together, the overexpression of CDC20 might be expected to lead to accelerated proliferation of cells, and the specific knockdown of CDC20 by siRNA did, in fact, show an inhibitory effect against cell growth *in vitro*.

The accurate transition from the S phase to the G2/M phase is crucial for the control of eukaryotic cell proliferation (31). It was previously reported that in metaphase to anaphase, APC/C-Cdc20 mediates the ubiquitination of securin and cyclin B1, allowing the activation of separase and the onset of anaphase and mitotic exit (18). CDC20 plays an indispensable role during the metaphase-to-anaphase transition by targeting critical cell cycle regulators including securin and cyclin B1 for ubiquitination-mediated destruction (19, 32, 33). Cyclin A was essential for the control of the cell cycle at the G1/S and the G2/M transitions (20, 34). In mitosis, it may contribute to the control of cyclin B1 stability (35). In this study, we found that the securin, cyclin B1, and securin protein levels were regulated by high expression of CDC20.

In conclusion, our study demonstrated the high expression of CDC20 involvement in tumorigenesis in WT. Functional experiments verified that suppression of CDC20 could inhibit WT cell proliferation, migration, and arrested cell cycle in G2/M phase. However, more underlying molecular mechanisms upstream of CDC20 still need further research. What is more, our study has limitations on the WT cell model such that G401 and SK-NEP-1 cells were formerly classified as WT cell lines, but they have since had more correct classifications (36). Overall, this finding provides a new focus that CDC20 may be a clinically relevant indicator and a promising therapeutic target of WT.

## Data Availability Statement

The original contributions presented in the study are included in the article/supplementary material, further inquiries can be directed to the corresponding author/s.

## Ethics Statement

The studies involving human participants were reviewed and approved by Children Hospital of Chongqing Medical University. Written informed consent to participate in this study was provided by the participants' legal guardian/next of kin.

## Author Contributions

QS, BT, DH, and GW conceived of and designed the study. BT, QS, YaL, and YoL made contributions in acquisition, analysis of the data, and drafted the manuscript. BT, QS, and YoL completed the experiments. DH, TL, and GW helped draft the manuscript and assisted in the analysis of the data. All authors contributed to the article and approved the submitted version.

## Conflict of Interest

The authors declare that the research was conducted in the absence of any commercial or financial relationships that could be construed as a potential conflict of interest.

## Publisher's Note

All claims expressed in this article are solely those of the authors and do not necessarily represent those of their affiliated organizations, or those of the publisher, the editors and the reviewers. Any product that may be evaluated in this article, or claim that may be made by its manufacturer, is not guaranteed or endorsed by the publisher.
